# Label-Free Imaging of Solid-Phase Peptide Synthesis Products and Their Modifications Tethered in Microspots Using Time-of-Flight Secondary Ion Mass Spectrometry

**DOI:** 10.3390/ijms242115945

**Published:** 2023-11-03

**Authors:** Dimitry Schmidt, Josef Maier, Hubert Bernauer, Alexander Nesterov-Mueller

**Affiliations:** 1 Institute of Microstructure Technology, Karlsruhe Institute for Technology, Hermann-von-Helmholtz-Platz 1, 76344 Eggenstein-Leopoldshafen, Germany; dimitry.schmidt@kit.edu; 2 ATG:biosynthetics GmbH, Weberstraße 40, 79249 Merzhausen, Germany; josef.maier@atg-biosynthetics.de (J.M.); hubert.bernauer@atg-biosynthetics.de (H.B.)

**Keywords:** imaging of chemical reactions, high-density peptide arrays, time-of-flight secondary ion mass spectrometry

## Abstract

Time-of-flight secondary ion mass spectrometry is used to analyze solid-phase synthesis products in 60 µm spots of high-density peptide arrays. As a result, a table of specific fragments for the individual detection of amino acids and their side chain protecting groups within peptides is compiled. The specific signal of an amino acid increases linearly as its number increases in the immobilized peptide. Mass-to-charge ratio values are identified that can distinguish between isomers such as leucine and isoleucine. The accessibility of the N-terminus of polyalanine will be studied depending on the number of its residues. The examples provided in the study demonstrate the significant potential of time-of-flight secondary ion mass spectrometry for high-throughput screening of functional groups and their accessibility to chemical reactions occurring simultaneously in hundreds of thousands of microreactors on a single microscope slide.

## 1. Introduction

### 1.1. Advancements in Solid-Phase Synthesis

The invention of Merrifield solid-phase synthesis enabled the development of DNA and peptide microarrays [1], which have become an important tool for the high-throughput study of oligonucleotide and protein interactions [2,3,4,5]. Today, the technical possibilities for the synthesis of molecular microarrays have been significantly expanded due to the addressing of monomers for in situ solid-phase synthesis using a xerographic printer [6], electric fields of a computer chip [7,8], light-induced forward transfer [9], and the stochastic filling of microcavities with microparticles [10]. In addition, the molecular space of microarrays increased significantly due to the use of post-synthesis modification of peptides [11,12,13], the introduction of hundreds of new artificial amino acids [14], and the implementation of peptoid chemistry in array format [15]. Such progress has enabled screenings for both a large number of new functional molecules and new efficient chemical reactions. To realize this possibility, the rapid imaging of numerous small molecular compounds covalently bound in ordered microspots is required.

### 1.2. Array-Based Label-Free Molecular Imaging

Array-based label-free molecular imaging is a valuable technique for studying molecular interactions without the need for fluorescent or radioactive labels. There are several methods for label-free molecular imaging in the high-density array format. Surface plasmon resonance (SPR) is a widely used label-free technique for the real-time monitoring of biomolecular interactions. It measures changes in the refractive index at the sensor surface as a result of binding events [16]. SPR relies on changes in refractive index occurring at the sensor surface when molecules bind to it. The molecules with a low molecular weight may not cause a significant change in the refractive index. As a result, their binding to the surface might not produce a detectable signal, making it challenging to study them using SPR.

Atomic force microscopy (AFM) is a high-resolution imaging technique that can be used to visualize different molecules on the same substrate at the nanoscale [17,18]. By functionalizing the AFM tip and substrate with the molecules of interest, binding molecular events can be directly observed. However, AFM imaging can exert physical forces on the sample during scanning, potentially leading to sample damage or alteration, especially when dealing with small compounds [19]. In addition, the correct interpretation of molecular imaging using AFM requires an understanding of the mechanisms of interaction of the functionalized type with molecules on the substrate.

Interferometric methods, such as white light interferometry, can be used to measure changes in optical path length, resulting from depositing the molecules on the array surface [20]. By immobilizing peptides on a substrate, changes in interference patterns can be detected upon binding events [21]. Interferometry is primarily designed for reflective surfaces. Despite the possibility of the highly sensitive detection of molecular accumulation on individual spots, on-reflective or transparent samples may require additional coatings or modifications to be suitable for measurement. While interferometry excels in measuring 2D surface profiles, it is less suited for full 3D topography measurements, such as on relatively thick polymer layers, on which peptide solid-phase synthesis occurs [22,23].

Against this background, time-of-flight secondary ion mass spectrometry (ToF-SIMS), a surface analysis technique first described by Benninghoven in 1994, seems promising for imaging molecular arrays [24].

### 1.3. Time-of-Flight Secondary Ion Mass Spectrometry for Molecular Imaging

ToF-SIMS allows for high lateral resolution, high chemical and surface selectivity, and sufficient mass resolution for the separation of major mass interferences [22,25]. The largest advantage of ToF-SIMS for evaluating solid-phase synthesis products and their post-synthesis modifications is its ability to detect low amounts of adsorbed peptides or proteins on a wide variety of surfaces [26]. ToF-SIMS was applied for monitoring peptides during the solid-phase synthesis on resin beads [27,28]. The primary objective of these studies was to identify the most suitable linker for facilitating the release of intact amino acid sequences (Boc-Pro-Phe-Leu) from the support during ToF-SIMS with Ga or In primary ions, while minimizing any potential fragmentation of the peptides.

The ToF-SIMS was utilized to detect proteins on a photolithographically patterned poly(ethylene glycol)-based polymer film [29]. This study compared the impact of different primary ion sources (Bi_1_^+^, Bi_3_^+^, and C_60_^+^) on the fragmentation pattern, surface sensitivity, and image contrast in ion images. The results show that Bi_3_^+^ provides better surface sensitivity and image contrast, particularly for low-mass fragments. The C_60_^+^ ion source was successfully used to characterize peptide-doped trehalose thin films [30]. Different peptides with varying hydrophobicity levels were studied via ToF-SIMS in two classic MALDI matrices [31]. A trehalose–glycerol matrix was utilized to generate high-resolution ToF-SIMS images of macrophages and glial cells [32].

These and other studies have paved the way for modern ToF-SIMS bioimaging [33,34,35], where not only peptides are detected, but also proteins, RNA, lipids, saccharides, and other biomolecules [36]. The most promising areas in ToF-SIMS imaging for life include the integration of ToF-SIMS into a multimodal setting, i.e., in combination with other imaging [37,38], as well as the use of machine learning methods to interpret spectral signals [39].

The ToF-SIMS imaging of the glycan and RNA microarray has been demonstrated to test the bioavailability of surface-bound biomolecules located in microspots [40,41]. This approach was limited to only two distinct pre-synthetized glycans and six DNAs, thereby preventing the full exploration of the ToF-SIMS screening capabilities for larger molecular libraries.

ToF-SIMS detection of small compounds on high-density molecular arrays has fundamental differences from bioimaging. This distinction lies in the fact that known molecules are positioned in specific spots on the array, such as known amino acid sequences in the case of peptides. Simultaneously, it is possible, through small yet varied modifications of these sequences, to thoroughly investigate the dependence of spectral signals on the oligomer’s length, its composition, and the presence of various protective groups. Addressing this issue, we evaluate the ToF-SIMS for the molecular imaging of solid-phase synthesis products using in situ synthetized high-density peptide microarrays.

## 2. Results

### 2.1. Homopeptides

**Figure 1:**
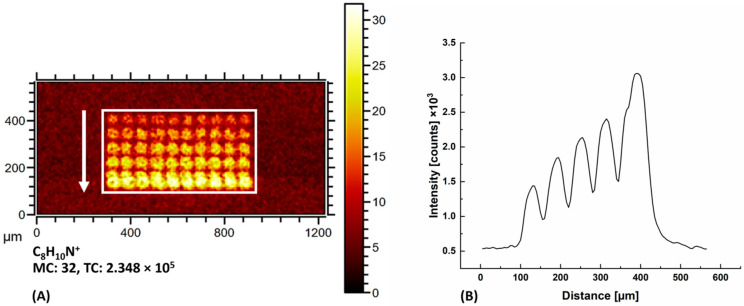
(**A**) Heatmap image of the characteristic phenylalanine ion, C_8_H_10_N^+^. Each of the ten identical columns includes five homopeptides with one to five residues (from the top to bottom). In order to minimalize the signal-to-noise ratio, all 10 columns have been measured. The white arrow shows the direction of increase in the intensity of the spots in the white box, the profile of which is shown in Figure 1B. (**B**) The vertical cross-section of the heatmap image in the white box.

Table 1 summarizes the specific ions and the corresponding mass-to-charge ratios identified for each of the twenty canonical amino acids and their side chain protecting groups in the respective polarity on the high-density peptide chip. We used these values for the imaging of homopeptide spots as shown in Figure 1. For example, in the case of the phenylalanine ion (C_8_H_10_N^+^) imaging, a linear increase in signal intensity was observed with an increase in the length of the homopeptide from one to five residues.

Most of the specific ions presented in Table 1 overlap with ions obtained from the detection of 16 proteinogenic amino acids in the composition of pre-synthesized, purified spin-coated peptides in the form of thin films on glass. [42]. However, the ToF-SIMS detection of the peptide spots synthetized on the chip revealed several peculiarities. There were overlaps in the spectra of some amino acids because ion bombardment formed identical fragments from different residues as in the case of amino acids arginine and proline. Nevertheless, all individual amino acids could be distinguished from each other using specific ions (see Table 1). This table also applies to amino acids if they have an attached side chain protecting group. The side chain protecting groups Boc (tert-butyloxycarbonyl) and tBu (tert-butyl) were very similar in structure and could only be detected as C_4_H_9_^+^.

**Figure 2:**
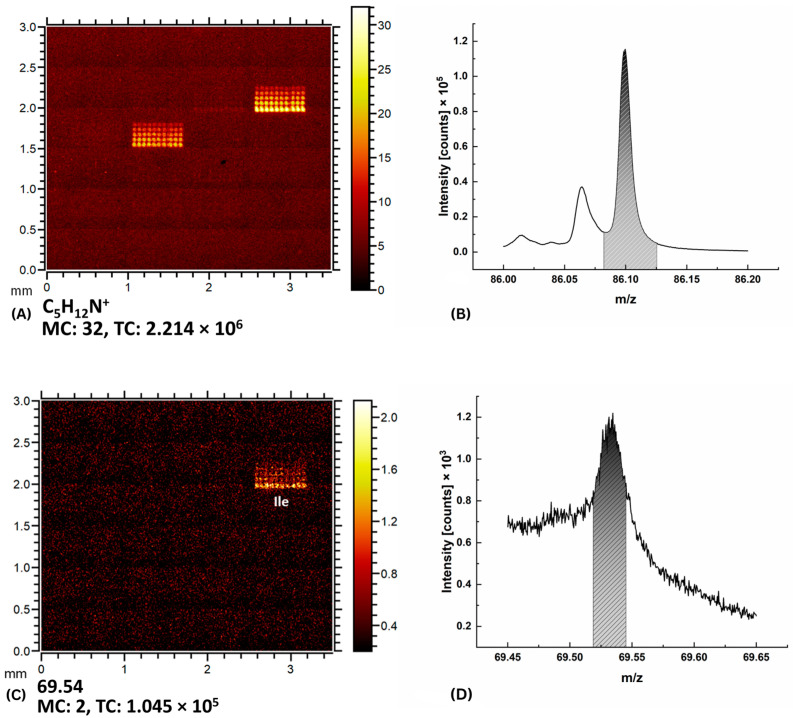
(**A**) Imaging of the section A1 for the C_5_H_12_N^+^ ion contained in both isoleucine Ile and leucine Leu. (**B**) Corresponding spectrum for C_5_H_12_N^+^. (**C**) Imaging of isoleucine Ile at 69.54 m/z. (**D**) The mass spectrum around the peak 69.54 m/z. Integrals over the selected area in the spectra in (**B**,**D**) are used to calculate the intensity of points when imaging dots in (**A**,**C**).

**Figure 3:**
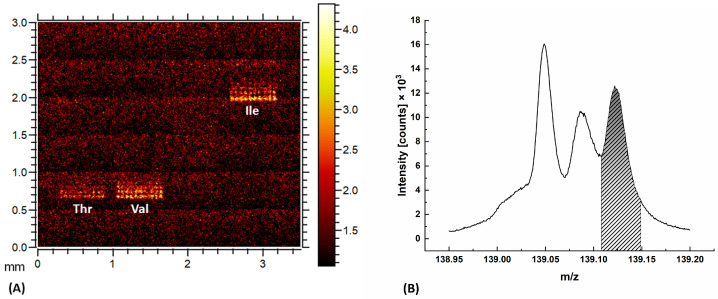
(**A**) The imaging of threonine Thr, valine Val, and isoleucine Ile with a mass peak of 139.12 m/z. (**B**) Mass spectrum of isoleucine at the mass peaks 139.05 m/z, 139.08 m/z, and 139.12 m/z.

Isoleucine and leucine shared several specific ions, for example, the ion C_5_H_12_N^+^ at the mass peak 86.1 m/z (Figure 2A,B). However, only isoleucine showed a signal at the mass peak of 69.54 m/z (Figure 2C,D) with significant contrast to the substrate. Isoleucine was also detected as a double-charged fragment at 69.54 m/z * 2 ≈ 139.1 m/z (Figure 3). The intensity of the signal at the mass peak 139.1 m/z was about 1/3 of the intensity of the signal at the mass peak of 69.54 m/z and could be used to reliably distinguish between two isomers. The chemical assignments of this signal are unknown. However, the signal at 139.1 m/z is not specific to isoleucine. It can also be utilized to visualize threonine and valine, as demonstrated in the heatmap in Figure 3. It is worth noticing that threonine and valine did not exhibit a signal at 69.5 m/z, as illustrated in Figure 2C.

Although β-alanine has the same mass as the canonical amino acid alanine, it could be selectively detected by the C_3_H_6_NO^+^ ion. However, β-alanine showed a strong crosstalk with the polymer layer.

The smallest amino acid glycine could not be detected with positive ions. For example, the possible CH_4_N^+^ ion showed no contrast between the glycine and the polymeric background. Unlike positive polarity, glycine was specifically detectable in negative polarity, with a high contrast at 73.03 m/z with C_2_H_3_NO_2_^−^.

### 2.2. Combinatorially Assembled Peptides

**Figure 4:**
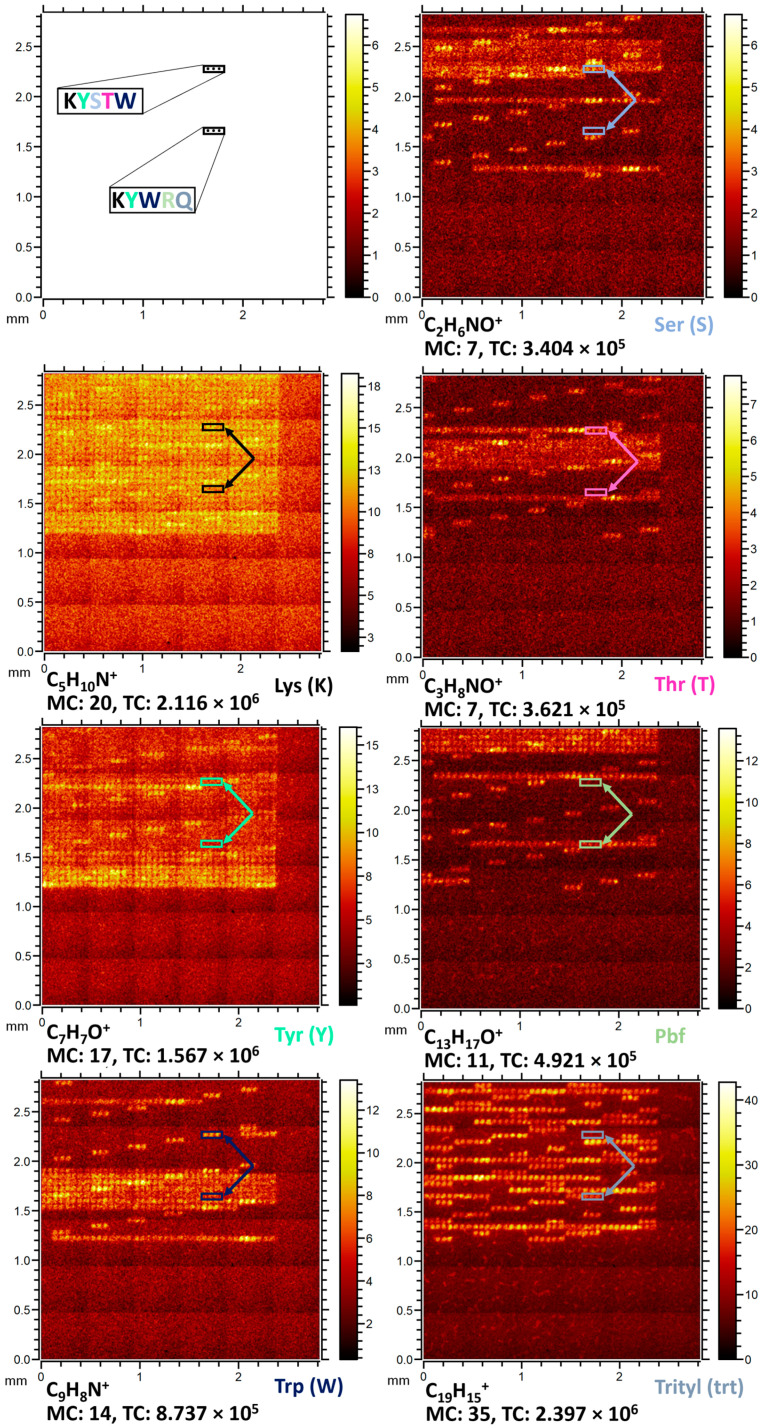
Imaging of peptides in section B2. Lysine (Lys) and tyrosine (Tyr) were shared by all peptides, while the remaining three amino acids serine (Ser), arginine (Arg) and tryptophan (Trp) varied combinatorially. All peptides were synthesized in triplicate spots. The frames highlight two peptides KYSTW and KYWRQ. Each heat map shows the characteristic ion signals for each amino acid and the corresponding protecting group Pbf (attached to arginine) and Trityl (attached to glutamine) within these peptides. Different colors of frames and arrows indicate the correspondingly colored amino acids in the subfigure in the upper left corner.

The amino acids were divided into two groups of those with protecting groups and those without. For each group, fully combinatorial 3-mer to 5-mer peptides were synthetized and analyzed via ToF-SIMS. The corresponding specific ions of the amino acids from the homopeptides were used to detect these amino acids inside the non-homogenous peptide under the same mass scale calibration (Figure 4). Figure 4 shows a fragment of the B2 section. The peptides synthesized in this section are of increased complexity with up to five different amino acids as well as their protection groups. We could detect single amino acids and the protecting groups Pbf (2,2,4,6,7-pentamethyl-dihydrobenzofuran-5-sulfonyl), Trt (triphenylmethyl), tBu (tert-butyl), and Boc (Tert-Butyloxycarbonyl) attached to the side chains inside the peptides as well.

### 2.3. The Influence of the Peptide Length on the ToF-SIMS Signal

**Figure 5:**
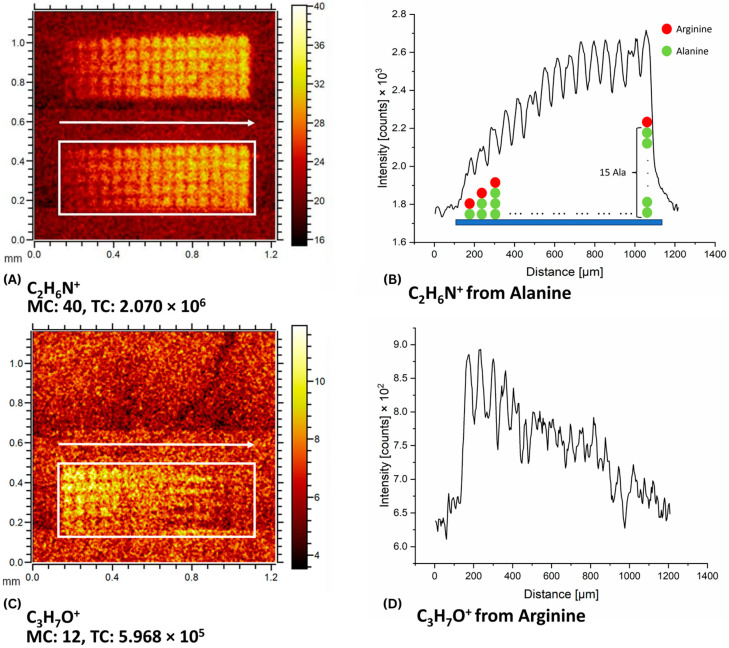
(**A**) Imaging of C_2_H_6_N^+^ ion for polyalanines as 1-mers to 15-mers from left to right per row aligned in two blocks. The white box in the heatmap shows the area along which the intensity of C_2_H_6_N^+^ has been measured. For a better signal-to-noise ratio, all 5 rows have been measured. The white arrow shows the direction of change in the intensity of the spots in the white rectangle, the profile of which is shown in Figure 5B. (**B**) Corresponding intensity graph of alanine measured along the indicated white frame. Schematic peptides placed on a blue substrate under the intensity curve correspond to local intensity peaks. Green spheres represent alanine and red spheres represent arginine in the peptide chain. (**C**) Imaging of C_3_H_7_O^+^ for arginine attached to the N-terminus of the polyalanines. The white arrow shows the direction of change in the intensity of the spots in the white rectangle, the profile of which is shown in Figure 5D. (**D**) Corresponding intensity graph of arginine measured along the indicated white frame in Figure 5C.

Polyalanines with a length of 1 to 15 residues were synthetized (five replicates for each peptide) and functionalized with an arginine as a head group (Figure 5). The imaging of polyalanine revealed a clear correlation with the number of residues that had been already observed in Section 2.1 for pentapeptides (Figure 5A). The C_2_H_6_N^+^ signal intensity of alanine was measured along the growing sequences. The intensity initially increased linearly and saturated from the ninth residue (Figure 5B). For arginine, a decrease in its specific ion signal intensity with every additional alanine residue was observed (Figure 5C,D). Such behavior of the signal for polyalanine may indicate the formation of a complex molecular peptide structure, in which the N-terminus is becoming more difficult to access for peptide bonds with the next residue, thus inhibiting the peptide chain growth and ultimately the attachment of the final arginine. This is probably due to the formation of self-associated β-sheet secondary structures by polyalanines, which remain stable even when the N-terminus of polyalanine is modified [43]. The trend toward a reduced N-terminal availability is more pronounced for arginine, one of the largest amino acids with a protecting group attached to its side chain.

## 3. Discussion

ToF-SIMS has a number of properties that make it attractive for screening functional groups as well as products of solid-phase synthesis in an array format with a high density of microspots.

Screening a 3 mm × 3 mm area with a lateral resolution of 5 µm takes approximately 15 min, which corresponds to a scanning velocity of <3 h/cm^2^. It would be possible to have a complete mass spectrum for 40,000 different products on one square centimeter at a spot pitch of 50 µm during this time. In addition, the liquid metal ion gun can provide a highly focused ion beam with dimensions of <50 nm [44]. Thus, the limitation on the lateral resolution of ToF-SIMS imaging lies with high-density molecular arrays and not with ToF-SIMS detection.

The detection limits of the ToF-SIMS for biological compounds are typically in the range of 30–3000 fmol/cm^2^ [45]. Consequently, it is capable of effectively detecting solid-phase synthesis products that are generated on the chip surface, even at a relatively small amount of approximately 200 pmol/cm^2^ [46].

One of the decisive advantages of ToF-SIMS for imaging molecular libraries is that no additional labels are needed for detection. Due to the wide mass spectrum, specific ions can be found that could ensure the detection of the desired molecule. This is especially beneficial for screening low-molecular-weight and chemically unstable compounds that cannot be functionalized with fluorescent labels. These compounds could be, for example, unmodified native antibiotics, for which a peptide binder is sought for the development of a diagnostic device.

Since we could distinguish amino acids with attached groups, the protecting groups themselves, and amino acids after deprotection, this makes it possible to screen chemical reactions at various stages, as well as non-specific interactions of small compounds, for example, with protein fragments on a high-density peptide chip.

The origin of some specific ions that we have identified, for example, in the case of glycine or beta-alanine, could not always be derived directly from the mass formula of the molecule, although they allow for its detection. In the case of screening of given compounds, additional studies are needed. For a more detailed understanding of the formation of secondary ions and sensitivity optimization, ToF-SIMS offers different primary ions besides bismuth, such as gallium, gold, indium, and metal alloys [47]. The choice of primary ions can be optimized in terms of the detection of electropositive or electronegative compounds [48]. We found that isoleucine can share the characteristic ions for detection both with its isomer and with other amino acids. This is a prerequisite for selective detection, where unambiguous detection will be achieved not through individual ions, but through a set of them, thus forming a detection code.

ToF-SIMS allows for detecting correlations between the characteristic secondary ion signal of a certain functional group and structure of the molecule where it is integrated. Thus, it is possible to draw conclusions about the availability of functional groups for synthesis, as in the example of the N-terminal of the polyalanine.

## 4. Materials and Methods

### 4.1. ToF-SIMS

**Figure 6:**
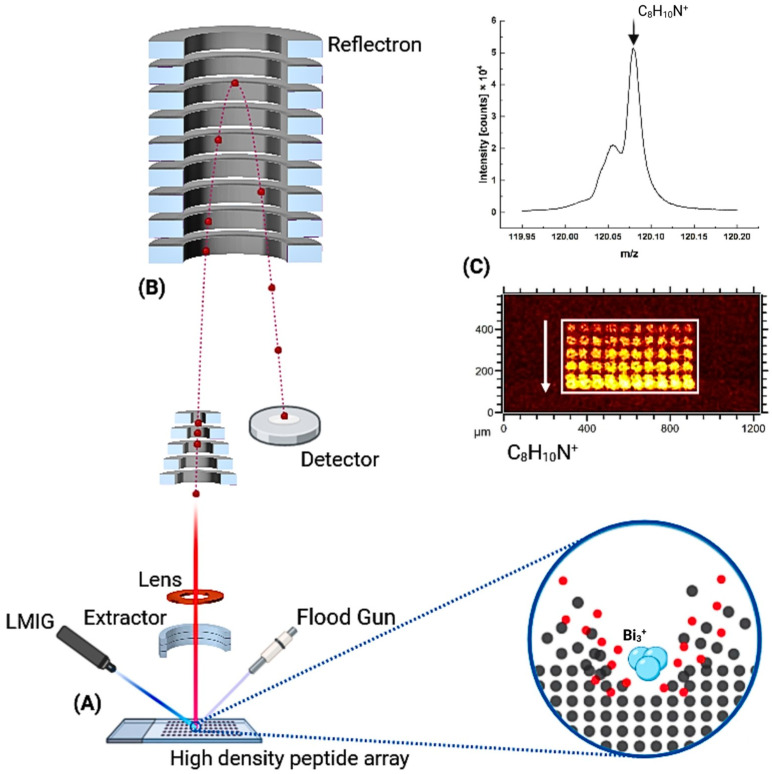
The ToF-SIMS system for imaging the molecular microspots arranged on the chip surface. (**A**) Generation of secondary ions (red dots) by bombardment with bismuth ions (Bi_3_^+^) from a LMIG (liquid metal ion gun). Gray dots indicate uncharged fragments of the chip surface. A flood gun is added to the system for charge compensation. (**B**) Generated secondary ions are collected by the extractor and pass the drift tube and the reflectron. (**C**) Mass spectrum and the imaging of the selected secondary ion C_8_H_10_N^+^. Spot size is 60 µm. The white arrow shows the direction of increase in the intensity of the spots in the white box.

Figure 6 illustrates the principle of the ToF-SIMS system, along with the corresponding mass spectra and images. The images represent a heat map depicting the spatial distribution of peptide microspots and the concentration of the corresponding molecular products obtained through solid-phase synthesis. The mass spectra were measured with the ToF-SIMS 5 system (IONTOF, Münster, Germany). A Bi-cluster liquid metal ion gun (Bi-cluster LMIG) generated Bi_3_^+^ primary ion pulses at 25 keV at 65 µs cycle time. A pulsed electron flood gun compensated sample charging. Short primary ion pulses (bunched mode) enabled the highest mass resolution. The chip surface was scanned over 3500 µm × 3500 µm with a lateral resolution of 5 µm. The mass spectra were calibrated with hydrocarbon fragments CH^+^, CH_2_^+^, C_2_H_3_^+^, C_6_H_3_^+^, C_6_H_4_^+^, C_6_H_5_^+^ in positive polarity and CH^−^, OH^−^, O_2_^−^, CO_2_^−^, C_4_H^−^ in negative polarity and analyzed with Surfacelab 7 [22].

### 4.2. Peptide Chip Design

**Figure 7:**
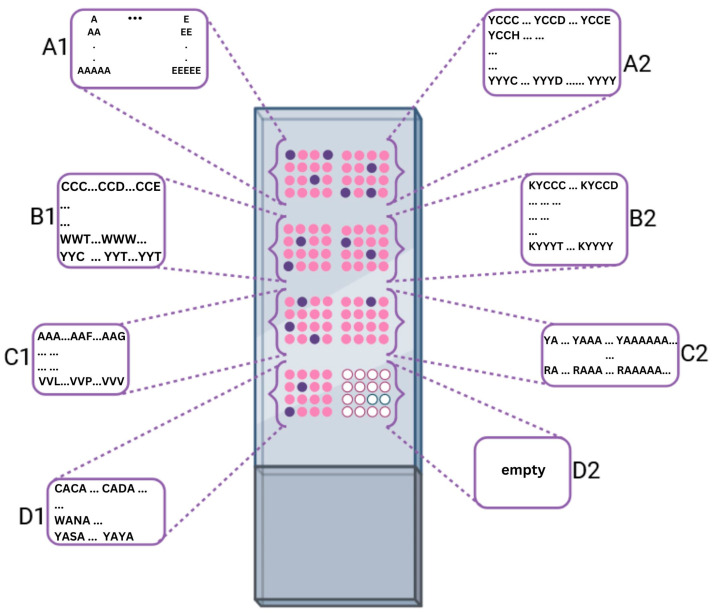
Layout of the slide. The slide is divided into 7 sections. The letters in the descriptive boxes schematically indicate the different types of peptide variations distributed across the sectors of the chip. Section A1 contains all 20 canonical amino acids, arranged in ascending order line by line from a 1-mer to a 5-mer, in 10 copies in blocks. Section A2 contains permutations of 4-mer peptides constructed from amino acids containing protecting groups. These are cysteine, aspartic acid, glutamic acid, histidine, lysine, asparagine, glutamine, arginine, serine, threonine, tryptophan, and tyrosine. Tyrosine is synthesized throughout as the fixed end of the peptide. The peptides are present in triplicate. Section B1 contains permutations of the same amino acids as 3-mer peptides without tyrosine. Section B2 consists of permutations of the same amino acids as 5-mer peptides with lysine and tyrosine as fixed ends in 3 copies each. Section C1 consists of permutations of the remaining amino acids without protecting groups as 3-mer peptides in 10 copies each. Section C2 consists of 2 blocks in which alanine is built up from a 1-mer to 15-mer peptide with tyrosine and arginine as fixed ends. Section D1 contains permutations of amino acids with a protecting group and alanine in between. Section D2 is empty.

The high-density peptide arrays (axxelera UG, Karlsruhe, Germany) with an area of 25.0 mm × 75.6 mm were synthetized on 3D amino-functionalized glass slides (PolyAn GmbH, Berlin, Germany). The peptide chip was partitioned into seven sections for time-of-flight secondary ion mass spectrometry (ToF-SIMS) analysis. The A1 section comprises blocks of homopeptides (Figure 7). Each block contains 5 rows of ten replica spots underneath each other with increasing number of amino acid residues (1–5 mer) (Figure 1A).

In addition to the 20 canonical amino acids, β-alanine was immobilized in the bottom left corner of section A1. The sections A2, B1, B2, C1, and D1 represent combinatorial combinations of different amino acids to investigate the influence of sequence specificity on the ToF-SIMS detection. Section C2 contains 15-mer amino acids to investigate the influence of the peptide length on the ToF-SIMS signal. Two identical chips were employed, where one chip maintained the protecting groups on the side chains, while the other chip underwent deprotection.

## 5. Conclusions

ToF-SIMS was demonstrated as a suitable technique to detect single amino acids within a peptide chain in a high-density array format. Therefore, we established a list of specific ions for each of the 20 canonical amino acids and different side chain protecting groups used in the solid-phase synthesis of peptides. It was shown that side chain protecting groups do not interfere with the detection of individual amino acids in peptide sequences. The ability to distinguish isomeric compounds via ToF-SIMS was shown. In our experiments, it was not possible to determine the position of the studied residue in the amino acid sequence. At the same time, significant differences were observed between peptides containing the same residue, but in different amounts. The effect of polyalanine length on the intensity of the specific ion signal of a single alanine and its modification with arginine as the head group was studied.

Due to the generation of a large number of signals from spots with known sequences, a well-labelled database arises for constructing machine learning algorithms in order to study the efficiency of the synthesis of various peptide chains, as well as study the interaction of peptides with other small compounds.

## Figures and Tables

**Table 1:** Characteristic ToF-SIMS signals for all 20 canonical amino acids. For each amino acid, the polarity of the signal and the corresponding mass-to-charge ratios and ions are indicated.

Name	Polarity	Mass-to-Charge Ratio	Ion
Alanine	Positive	44.05	C_2_H_6_N^+^
	Negative	87.06	C_3_H_5_NO_2_^−^
Cysteine	Positive	44.98 46.99 56.98 58.99 76.0 121.0	CHS^+^ CH_3_S^+^ C_2_HS^+^ C_2_H_3_S^+^ C_2_H_6_SN^+^ C_3_H_7_SNO_2_^+^
	Negative	31.97 32.98 56.98 70.98 99.986	S^−^ HS^−^ C_2_HS^−^ C_2_HSN^−^ C_3_H_2_SNO^−^
Aspartic Acid	Positive	88.04	C_3_H_6_NO_2_^+^
	Negative	40.02 70.03 96.01	C_2_H_2_N^−^ C_3_H_4_NO^−^ C_4_H_2_NO_2_^−^
Glutamic Acid	Positive	84.046 10205	C_4_H_6_NO^+^ C_4_H_8_NO_2_^+^
	Negative	54.04 71.02 82.03 98.03 100.05 127.06	C_3_H_4_N^−^ C_3_H_3_O_2_^−^ C_4_H_4_NO^−^ C_4_H_4_NO_2_^−^ C_4_H_6_NO_2_^−^ C_5_H_7_N_2_O_2_^−^
Phenylalanine	Positive	91.05 103.05 120.08	C_7_H_7_^+^ C_8_H_7_^+^ C_8_H_10_N^+^
	Negative	71.02 91.06 116.05 118.07 146.07	C_3_HO_2_^−^ C_7_H_7_^−^ C_8_H_6_N^−^ C_8_H_8_N^−^ C_9_H_8_NO^−^
Glycine	Positive	30.03	CH_4_N^+^
	Negative	73.03	C_2_H_3_NO_2_^−^
Histidine	Positive	-	-
	Negative	65.01	C_3_HN_2_^−^
Isoleucine	Positive	69.54 86.10	No assignment C_5_H_12_N^+^
	Negative	82.07 129.08	C_5_H_8_N^−^ C_6_H_11_NO_2_^−^
Lysine	Positive	74.02 84.08	C_2_H_4_NO_2_^+^ C_5_H_10_N^+^
	Negative	-	-
Leucine	Positive	86.10	C_5_H_12_N^+^
	Negative	82.07 129.08	C_5_H_8_N^−^ C_6_H_11_NO_2_^−^
Methionine	Positive	44.98 46.99 48.0 61.01	CHS^+^ CH_3_S^+^ CH_4_S^+^ C_2_H_5_S^+^
	Negative	31.97 32.98 44.98 46.99 47.01 56.98 58.99	S^−^ HS^−^ CHS^−^ CH_3_S^−^ CH_3_O_2_^−^ C_2_HS^−^ C_2_H_3_S^−^
Asparagine	Positive	87.05	C_3_H_7_N_2_O^+^
	Negative	77.04 96.01	C_6_H_5_^−^ C_4_H_2_NO_2_^−^
Proline	Positive	68.05 70.07 71.07	C_4_H_6_N^+^ C_4_H_8_N^+^ C_4_H_9_N^+^
	Negative	-	-
Glutamine	Positive	101.08	C_4_H_9_N_2_O^+^
	Negative	77.05 127.05	C_6_H_5_^−^ C_4_H_2_NO_2_^−^
Arginine	Positive	59.05 60.06 70.07 73.06 87.08	C_3_H_7_O^+^ CH_6_N_3_^+^ C_4_H_8_N^+^ C_2_H_7_N_3_^+^ C_4_H_11_N_2_^+^
	Negative	40.0 41.01	CN_2_^−^ CHN_2_^−^
Serine	Positive	60.05	C_2_H_6_NO^+^
	Negative	-	-
Threonine	Positive	74.06	C_3_H_8_NO^+^
	Negative	-	-
Valine	Positive	72.08	C_4_H_10_N^+^
	Negative	-	-
Tryptophan	Positive	130.07	C_9_H_8_N^+^
	Negative	116.05 140.05 142.07	C_8_H_6_N^−^ C_10_H_6_N^−^ C_10_H_8_N^−^
Tyrosine	Positive	107.05 136.08 147.04	C_7_H_7_O^+^ C_8_H_10_NO^+^ C_9_H_7_O_2_^+^
	Negative	93.03 117.03 119.05 134.06	C_6_H_5_O^−^ C_8_H_5_O^−^ C_8_H_7_O^−^ C_8_H_8_NO^−^
β-Alanine	Positive	72.05	C_3_H_6_NO^+^
	Negative	58.03	C_2_H_4_NO^−^
Tert-Butyl	Positive	57.07	C_4_H_9_^+^
	Negative	15.02 57.04	CH_3_^−^ C_4_H_9_^−^
Triphenylmethyl	Positive	165.06 243.12	C_13_H_9_^+^ C_19_H_15_^+^
	Negative	-	-
Tert-Butyloxycarbonyl	Positive	57.07	C_4_H_9_^+^
	Negative	-	-
Pentamethyldihydrobenzofuransulfonyl	Positive	188.12	C_13_H_16_O^+^
	Negative	63.96 77.97 188.12 252.08	SO_2_^−^ CH_2_SO_2_^−^ C_13_H_16_O^−^ C_13_H_16_SO_3_^−^

## Data Availability

The data presented in this study are available on request from the corresponding author.
